# Letter to the editor: in response to Alstrup et. al.

**DOI:** 10.1186/s13049-019-0638-y

**Published:** 2019-06-17

**Authors:** Cor Slagt, Amon Heijne, Geert-Jan van Geffen

**Affiliations:** Department of Anesthesiology, Pain and Palliative care, Radboud University Medical Centre, PO box 9101, 6500 HB Nijmegen, The Netherlands

## Letter to the editor: in response to Alstrup et. al.

Dear Editor,

With great interest we have read the article written by Alstrup et al. [1] recently published in the Scandinavian Journal of Trauma, Resuscitation and Emergency medicine. They present the quality and potentials of their helicopter emergency medical service (HEMS) database. In the Netherlands the prehospital HEMS care is similarly organized as in Denmark. The country is divided in four HEMS regions of similar size, each operating its own digital registration.

As our operation has evolved into a 24/7 coverage in the last decade our database also had to develop and expand. Our most recent evolvement was extensive, such as documentation of prehospital ultrasound. To ensure each dispatch is documented properly, each record is reviewed by the other HEMS team member after completion and each week all records are reviewed by one of three chief physicians.

To optimize database data Arts and colleagues have identified causes with can lead to insufficient data registration at different stages of data collection and registration [2]. They also propose a framework of procedures to assure data quality with respect to prevention, detection and actions for quality control [2].

When the patient is delivered to the hospital and upon completing the database record, a concise handover is compiled from the database and digitally sent to the receiving hospital. Our database includes 23,616 dispatch calls and 11,784 patient records. For follow-up, we are dependent on the receiving hospitals. We were able to acquire outcome data on 25% of our patients. Apart from the legal obligation to keep a medical record for each patient treated, our database is available for medical research. The large numbers of dispatches and patient contacts, the scarcity of missing information and the extensive quality control of the prospective data resulted in numerous publications and a number of PhD-theses [3, 4].

We suggest that for maximal efficiency of these databases, outcome data should be included. These clinical databases are essential to medical care and offer an excellent way to improve the care we provide [5].

## Data Availability

Data sharing not applicable to this article as no datasets were generated or analyzed during the current study.
